# Effect of Baghdadite Substitution on the Physicochemical Properties of Brushite Cements

**DOI:** 10.3390/ma12101719

**Published:** 2019-05-27

**Authors:** Young Jung No, Ib Holzmeister, Zufu Lu, Shubham Prajapati, Jeffrey Shi, Uwe Gbureck, Hala Zreiqat

**Affiliations:** 1 Biomaterials and Tissue Engineering Research Unit, University of Sydney, Darlington 2006, Australia; zufu.lu@sydney.edu.au (Z.L.); spra0038@uni.sydney.edu.au (S.P.); 2 Department for Functional Materials in Medicine and Dentistry, University of Würzburg, 97070 Würzburg, Germany; ib.holzmeister@fmz.uni-wuerzburg.de (I.H.); uwe.gbureck@fmz.uni-wuerzburg.de (U.G.); 3 School of Chemical Engineering, University of Sydney, Darlington 2006 Australia; jeff.shi@sydney.edu.au

**Keywords:** baghdadite, calcium phosphate cement, radiopacity, setting reaction, mechanical performance

## Abstract

Brushite cements have been clinically used for irregular bone defect filling applications, and various strategies have been previously reported to modify and improve their physicochemical properties such as strength and injectability. However, strategies to address other limitations of brushite cements such as low radiopacity or acidity without negatively impacting mechanical strength have not yet been reported. In this study, we report the effect of substituting the beta-tricalcium phosphate reactant in brushite cement with baghdadite (Ca_3_ZrSi_2_O_9_), a bioactive zirconium-doped calcium silicate ceramic, at various concentrations (0, 5, 10, 20, 30, 50, and 100 wt%) on the properties of the final brushite cement product. X-ray diffraction profiles indicate the dissolution of baghdadite during the cement reaction, without affecting the crystal structure of the precipitated brushite. EDX analysis shows that calcium is homogeneously distributed within the cement matrix, while zirconium and silicon form cluster-like aggregates with sizes ranging from few microns to more than 50 µm. X-ray images and µ-CT analysis indicate enhanced radiopacity with increased incorporation of baghdadite into brushite cement, with nearly a doubling of the aluminium equivalent thickness at 50 wt% baghdadite substitution. At the same time, compressive strength of brushite cement increased from 12.9 ± 3.1 MPa to 21.1 ± 4.1 MPa with 10 wt% baghdadite substitution. Culture medium conditioned with powdered brushite cement approached closer to physiological pH values when the cement is incorporated with increasing amounts of baghdadite (pH = 6.47 for pure brushite, pH = 7.02 for brushite with 20 wt% baghdadite substitution). Baghdadite substitution also influenced the ionic content in the culture medium, and subsequently affected the proliferative activity of primary human osteoblasts in vitro. This study indicates that baghdadite is a beneficial additive to enhance the radiopacity, mechanical performance and cytocompatibility of brushite cements.

## 1. Introduction

Bone cements are either based on polymeric polymethylmethacrylate (PMMA) formulations or on self-setting mixtures of calcium orthophosphate compounds. PMMA cements are mainly used in load-bearing applications due to their excellent mechanical properties, however, they are at the same time non-resorbable and remain as a permanent implant in the body [1]. In contrast, calcium phosphate cements (CPC) are less mechanically stable and have a brittle ceramic character, which limits their application to non-load-bearing application sites [2]. Cement setting occurs due to the different solubility of the cement raw materials and the final setting product by a continuous dissolution-precipitation reaction. Depending on the pH of the cement paste, either hydroxyapatite is formed at neutral or alkaline pH or the secondary phosphates brushite (CaHPO_4_·2H_2_O) or monetite (CaHPO_4_) are formed in more acidic conditions [3]. While hydroxyapatite has only a low solubility in vivo and can be only degraded by osteoclastic cells [4], brushite cements for bone defect filling applications have gathered increasing research attention recently due to their ability to partially resorb in vivo and support surrounding bone tissue growth [5,6]. In addition, strategies to overcome inherent issues of brushite cement such as injectability, rapid reaction, and lack of initial strength have been extensively investigated [7,8,9,10,11,12,13,14,15,16,17,18,19]. There is however limited literature on strategies addressing the issues regarding exothermicity of the cement reaction, acidity, and low radiopacity of brushite cements. The reaction between beta-tricalcium phosphate (β-TCP) and primary calcium phosphates in equimolar ratios to produce stoichiometric brushite is an exothermic acid-base reaction [20,21], and the cement paste causes the surrounding aqueous environment to become acidic [5,22]. Subsequently, the acidic reaction can detrimentally impact host bone tissue response [23,24], especially upon injection. In addition, the use of certain setting retardants and modifiers to control injectability, setting time, and strength have been shown to detrimentally impact cytocompatibility [13,25] and the ability to be resorbed in vivo [26].

Radiopacity of bone cements is also an important property to consider when monitoring the cement and the surrounding bone tissue during and after surgery. The radiopacity of a material is a function of the x-ray mass attenuation coefficients of the elements in its composition, with generally higher values with heavier elements, and density of the material, given that the x-ray mass attenuation coefficient of water is negligible in the context of inorganic materials such as cements and ceramics [27]. The elemental composition of calcium phosphate-based cements such as brushite is similar to human bone, hence the ability to adequately distinguish both under conventional x-ray imaging is difficult. Current strategies to enhance radiopacity of bone cements, both acrylic-based and calcium phosphate-based, use hard bioinert particles with high radiopacity such as barium sulfate and zirconia that are simply embedded into the brittle cement structure [28,29]. These embedded radiopaque particles have been found to act as stress concentration points and subsequently weaken the mechanical properties of the cement [28,30].

Baghdadite (Ca_3_ZrSi_2_O_9_) is a zirconium-incorporated calcium silicate ceramic that has been previously shown to support and enhance bone tissue ingrowth both in vitro and in animal in vivo models in its sintered scaffold form when compared to calcium phosphate ceramics [31,32,33,34]. The presence of the heavy element zirconium results in baghdadite possessing a higher X-ray mass attenuation coefficient compared to other ceramics for bone repair such as calcium phosphate, calcium sulfate, and calcium silicate. Baghdadite microparticles have previously been incorporated into polymer composites to enhance their strength, radiopacity, and bioactivity [35]. Similar to tricalcium phosphate, baghdadite is also slightly soluble in aqueous physiologically-relevant media [31,32], and hence has the potential to release calcium ions, which are also thought to form brushite in the presence of an acidic phosphate source. Here, we hypothesize that through the novel approach of substituting of beta-tricalcium phosphate with baghdadite in a brushite cement formulation, we would be able to synthesize brushite cements with enhanced radiopacity, while maintaining biocompatibility of the cement samples. This approach is novel, since the baghdadite is not simply a radiopaque filler in the cement matrix but acts as further cement reactant due to its calcium ion release. This will likely overcome the above described limitations of weakening the mechanical performance of the cement by chemically binding the particles within the cement matrix. This study investigates the effect of the substitution of β-TCP with baghdadite at various concentrations (0, 5, 10, 20, 30, 50, 100 wt% of β-TCP) on the brushite cement reaction and the physicochemical properties of set brushite cement. 

## 2. Materials and Methods 

### 2.1. Synthesis of Brushite Cement and Baghdadite-substituted Brushite Cement

Beta-tricalcium phosphate (β-TCP; β-Ca_3_(PO_4_)_2_) was synthesized by heating a mixture of Dicalcium phosphate anhydrous (CaHPO_4_, Mallinckrod-Baker, Phillipsburg, USA) and calcium carbonate (Merck, Darmstadt, Germany) in a 2:1 ratio to 1100 °C for 5 h. The sintering cake was crushed and ball-milled for 1 h with a planetary ball-mill (PM400, Retsch, Haan, Germany) to a final medium particle size d_50_ of 14.5 µm. Baghdadite (Ca_3_ZrSi_2_O_9_,) powder was synthesized by mixing zirconium oxide (45.2 g, Sigma, Darmstadt, Germany), calcium carbonate (122.3 g, Merck, Germany), and silica (49.3 g, Sigma, Germany) for 2 h in a ball mill (Retsch PM400, Haan, Germany). The powder mixture was sintered at 1300 °C for 3 h, followed by manually crushing and ball-milling for 30 min to obtain a particle size d_50_ of approximately 8.1 µm. Unmodified brushite cement (BC) samples were prepared by mixing equimolar amounts of β-TCP and monocalcium phosphate anhydrous (Ca(H_2_PO_4_)_2_), MCPA, Budenheim, Germany; d_50_ = 21.1 µm); for the cement samples modified with baghdadite, the β-TCP reactant was subsequently replaced with baghdadite i.e., 5 wt%, 10 wt%, 20 wt%, 30 wt%, 50 wt%, and 100 wt%, and the final cements were labelled as BCB5, BCB10, BCB20, BCB30, BCB50, and BCB100, respectively, as detailed in Table 1. Freshly prepared 0.1 M sodium pyrophosphate (NaPP, Sigma Aldrich) solution was added to the reaction mixture as a setting retardant in order to increase the handling period without negatively affecting its cytocompatibility [13]. The powders were mixed with the NaPP solution at a powder-to-liquid ratio of 3.0 g/mL on a glass slab using a spatula, poured into appropriate molds for cement characterization, and allowed to set at 37 °C and 100% relative humidity for 24 h. 

### 2.2. X-ray Diffraction Analysis of Brushite and Baghdadite-Incorporated Brushite Cements

Brushite and baghdadite-substituted cement pastes were poured into cylindrical molds 15 mm diameter and 10 mm thickness and left to set for 30 min for initial setting. The initially set cements were then removed from their molds and immersed in Milli-Q deionized water for one day at 37 °C for further setting. The disks were then dried at 40 °C and crushed into powder, and the x-ray diffraction (XRD) profiles for the powdered cement samples were obtained using and x-ray diffractometer (D5005, Siemens, Berlin, Germany) in a 2Theta range of 10–50° and a step size of 0.01°. The obtained XRD pattern profiles were compared to International Centre for Diffraction Data powdered diffraction file (ICDD-PDF) database. To identify possible changes in the crystal lattice of the brushite setting product, a second set of powders with the addition of 20% crystalline alpha-corundum (Baikowski, Regular CR1) as internal standard was measured and changes of the lattice parameters were calculated based on Rietveld refinement analysis with the software Topas 2.0 (Siemens, Berlin, Germany). 

### 2.3. Compressive Mechanical Properties

For compressive testing, brushite and baghdadite-substituted brushite cement pastes were poured into rectangular molds of 6 × 6 × 12 mm. After 30 min of initial setting at room temperature, the set cement was removed from the cylinders with the molds, were then immersed in deionized water for one day at 37 °C. Compressive strength and modulus of 8–10 specimens per variation were measured and recorded using a universal testing machine (Zwick, Ulm, Germany), using a load cell of 10 kN, at a compression rate of 1 mm/min.

### 2.4. Microstructure and Radiopacity of Cements

Solid disks of set brushite and baghdadite-substituted brushite with a diameter of 20 mm and a height of 2 mm were prepared for radiopacity analysis by pouring the cement pastes into smooth silicon rubber molds, left to set for 30 min, then removed from the molds and subsequently immersed in milliQ deionized water for one day at 37 °C for further setting. For radiopacity, the solid cement disks with a thickness of 2.0 mm were scanned using a dental X-ray machine (Heliodent DS, Sirona, Germany) at x-ray energy of 70 keV together with an aluminum stair with a step size ranging 0.5–5.0 mm in 0.5 mm steps. The greyscales of the samples in the obtained images were calculated using image analysis software (ImageJ), and were compared to the greyscales of the aluminum stair to calculate the aluminum equivalent thickness according to ISO 4049 [36]. In addition, the solid cement disks were scanned using microcomputed tomography (Xradia MicroXCT-400, Pleasanton, CA, USA) at x-ray energy of 70 keV. The greyscales of the samples in the obtained CT images were calculated using image analysis software (ImageJ), and were calibrated to the greyscales of air and water to obtain the Hounsfield units (HU) of each sample. According to definition, air is −1000 HU, whereas water has a value of 0 HU. For scanning electron microscopy, the hardened cement pieces from compressive strength testing were mounted onto stubs and the fracture surfaces were sputter coated with platinum, and subsequently examined under a scanning electron microscope (Zeiss CB 340, Carl Zeiss Microscopy GmbH, Oberkochen, Germany). Element maps for calcium, phosphorous, zirconium, and silicon were recorded with an attached energy-dispersive X-ray spectroscope (EDS) (XMaxN, Oxford-Instruments, Wiesbaden, Germany) and evaluated with the associated software AZtec (Oxford-Instruments, Wiesbaden, Germany). 

### 2.5. Investigating Primary Human Osteoblast Cytocompatibility

Based on the results of the previous experiments, BC, BCB5, BCB10, and BCB20 were chosen for assessment of cytocompatibility. The cytocompatibility of BC, BCB5, BCB10, and BCB20 on primary human osteoblasts (HOB) was investigated using cytotoxicity tests outlined in ISO/EN 10993-5 [37]. Permission to use discarded human tissue was granted by the Sydney Children’s Hospitals Network Human Research Ethics Committee (reference code: HREC/14/SCHN/36) and informed consent was obtained. Human trabecular bone was used for isolating HOB as described previously [35]. The cells were cultured at 37 °C with 5% CO_2_, and culture medium was changed every three days until cells were passaged at 80–90% confluence. All HOBs used in the experiments were at passage 2. Firstly, BC, BCB5, BCB10, and BCB20 cements were prepared, set in cylindrical molds of 15 mm diameter and 10 mm thickness, and left to further hydrate in deionized water at 37 °C for one day. The set cements were then removed and crushed using mortar and pestle. The cement powders were then immersed in serum-free α-MEM culture medium at a ratio of 200 mg in 1 mL culture medium and left to incubate at 37 °C for seven days. After incubation, the mixture was centrifuged to separate the culture medium and the cement powders. The extracted culture media were then sterilized by filtration (0.22 μm), and 10 *v*/*v*% of fetal bovine serum and 1 *v*/*v*% penicillin/streptomycin solution were added to make complete media and used for the HOB culture. The pH of the conditioned media was then obtained by isolating 5 mL of each conditioned media and recording its pH using a pH electrode, and the ionic content of the conditioned culture media was collected and analysed using inductively coupled plasma atomic emission spectroscopy (ICP-AES; Perkin Elmer, Optima 3000DV, Waltham, MA, USA).

HOB were seeded at a density of 1.0 × 10^3^ cells cm^−2^ into 48-well plates and incubated in 200 μL of prepared media for 24 h. Cells were then incubated at 37 °C in 5% CO_2_ for one and seven days. Viable cell numbers were evaluated at appropriate time intervals using the MTS assay (Promega, Madison, WI, USA) according to the manufacturer’s instructions. At the predetermined time point, the culture medium was removed, and then 100 μL of MTS assay solution was added to each well and was incubated for 4 h at 37 °C. The MTS assay produces an insoluble compound called formazan proportional to the number of live viable cells. The absorbance of the formazan was read at 492 nm using a microplate reader and graphed in Microsoft Excel. Four samples were used for each time point per cement group.

### 2.6. Statistical Analysis

All data are expressed as mean ± standard deviation (SD). For statistical analysis, Levene’s test was performed to determine the homogeneity of variance of data, and then either Tukey’s HSD or Tamhane’s post hoc tests were used. SPSS software (IBM) was used for all statistical analyses and differences were considered as significant if *p* < 0.05. 

## 3. Results

### 3.1. Phase Composition

**Figure 1:**
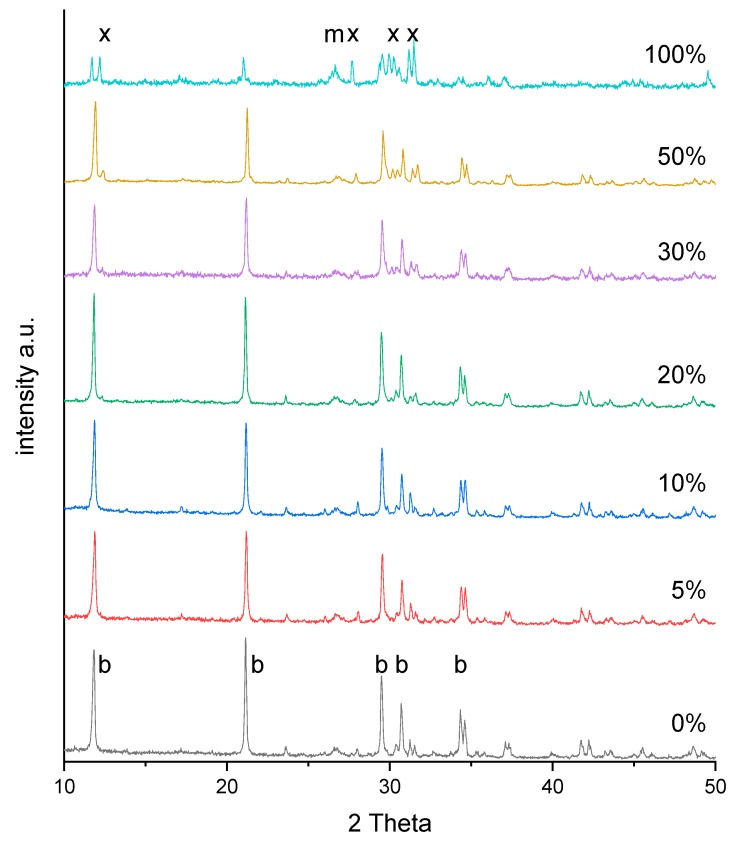
X-ray diffraction patterns of BCB samples with 0%, 10%, 20%, 30%, 50%, and 100% β-TCP substitution by baghdadite after one day setting in deionized water at 37 °C with marked peaks of brushite (b, ICDD-PDF #09-0077)), monetite (m, ICDD-PDF #09-0080), and baghdadite (x, ICDD-PDF #54-0710).

The XRD profiles between 2-theta angles of 10° and 50° for brushite and baghdadite-substituted brushite cements after one day setting in deionized water at 37 °C are shown in Figure 1. The XRD profiles for all cements showed distinct crystalline peaks corresponding to brushite (ICDD-PDF #09-0077) and a smaller fraction of monetite (CaHPO_4_, ICDD-PDF #09-0080). The crystalline peaks corresponding to baghdadite (ICDD-PDF #54-0710) were not reliably detected in the XRD profiles of brushite cements with less than 30 wt% substitution of baghdadite. The major crystalline peaks corresponding to baghdadite appeared in the XRD profile of BCB30 at very low relative intensities with respect to the crystalline peak of brushite corresponding to plane (020). For the BCB100 both the formation of brushite and monetite as well as a larger amount of unreacted baghdadite could be detected. To further analyze whether the substitution with baghdadite influences the lattice parameter of the precipitated brushite crystals, a diffraction pattern of the BCB50 sample with an added internal corundum standard was collected and the peak shift for different lattice planes was analyzed (Table 2). The results showed that the peak shift is overall marginal, which indicates that the further ions present in baghdadite (SiO_4_^4−^, Zr^4+^) are most likely not incorporated into the crystalline brushite phase and must have formed an amorphous fraction within the cement matrix due to the absence of further reflection peaks. 

### 3.2. Compressive Strength and Modulus

**Figure 2:**
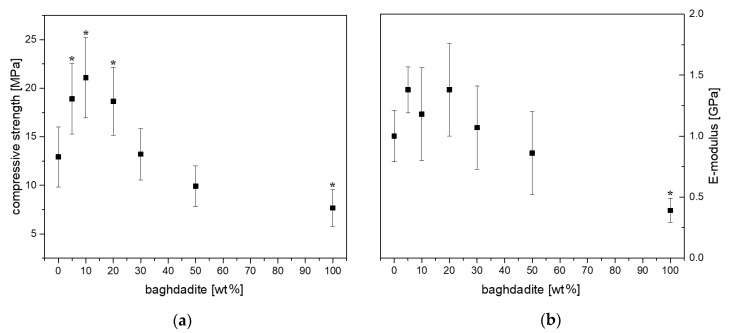
Compressive strength (**a**) and E-modulus (**b**) with different amount of baghdadite substitution of beta-TCP in brushite cement formulations. *: *p* < 0.05 versus pure set brushite cement.

Unmodified set brushite cement exhibited an average compressive strength of 12.9 ± 3.1 MPa and an E-modulus of 1.00 ± 0.21 GPa (Figure 2). Increased baghdadite substitution into brushite was shown to increase the compressive strength and modulus of the cement with statistical significance, with BCB10 exhibiting a compressive strength and modulus of 21.1 ± 4.1 MPa and 1.18 ± 0.38 GPa, respectively, and BCB100 exhibiting a compressive strength and modulus of 7.7 ± 1.9 MPa and 0.39 ± 0.1 GPa, respectively.

### 3.3. Microstructure under Scanning Electron Microscopy and Computed Tomography

**Figure 3:**
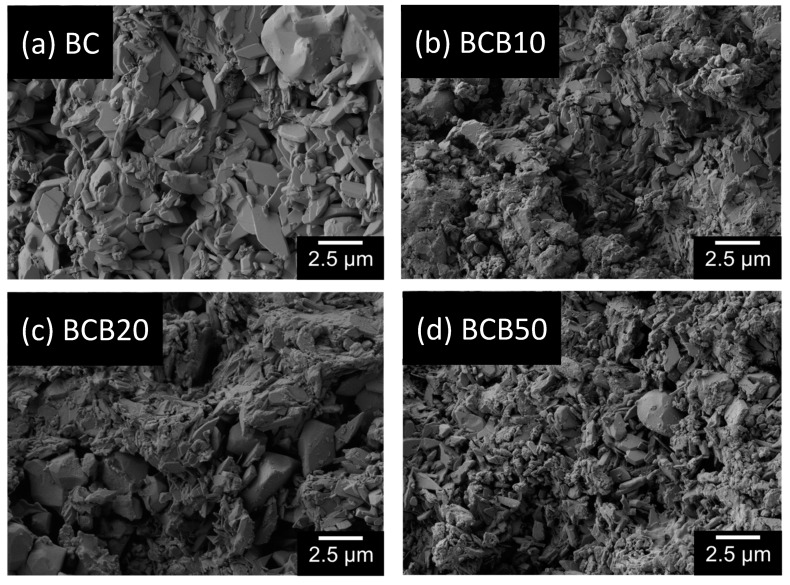
Scanning electron microscopy images of the cement fracture surfaces for (**a**) BC; (**b**) BCB10; (**c**) BCB20; and (**d**) BCB50.

**Figure 4:**
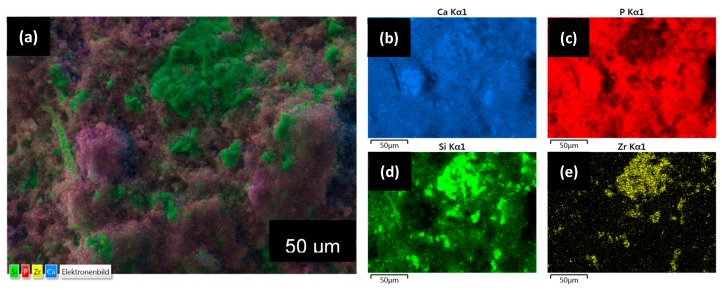
(**a**) Elemental mapping overlay image for BCB50; and distribution mapping of (**b**) calcium, (**c**) phosphorus, (**d**) silicon, and (**e**) zirconium within the BCB50 cement microstructure determined by EDX analysis.

**Figure 5:**
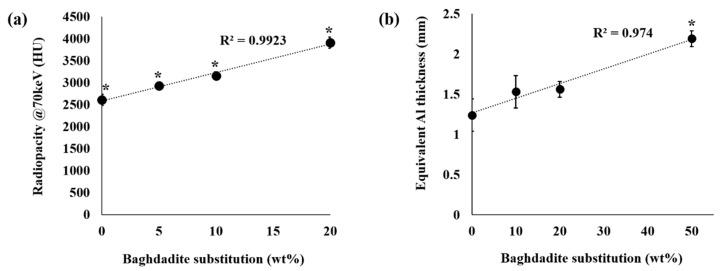
Graphs of the (**a**) micro-CT measured and (**b**) X-ray measured radiopacity of the brushite cement samples with increased baghdadite substitution; *: *p* < 0.05 vs. all other groups.

**Figure 6:**
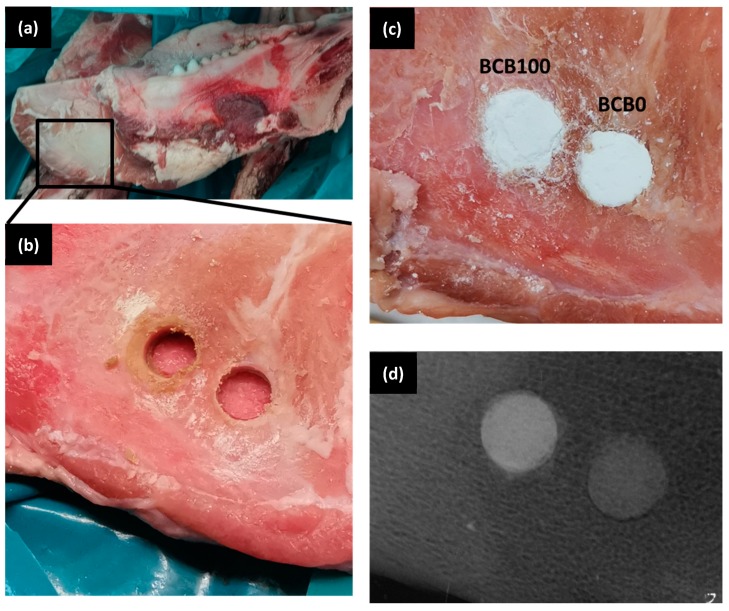
(**a**) Photographs of porcine cadaver mandible with (**b**) 6 × 3.5 mm bore holes generated with a trepan drill, and (**c**) filled with either BC (BCB0) or BCB100 cement. (**d**) An X-ray image of the cement implants demonstrates an improved visibility due to the higher radiopacity of the BCB100 cement.

Figure 3 shows the representative scanning electron microscopy images of BC, BCB10, BCB20, and BCB50. Irregular plate-like crystal structures can be observed for all cement groups throughout the cement fracture surface when observed using secondary electron detectors. With increasing baghdadite content, areas with agglomerates of sub-micron sized particles can be observed. When measuring the distribution of elements within the cement structure, it appears that calcium is homogeneously distributed within the matrix, while zirconium and silicon form cluster-like aggregates with sizes ranging from a few microns to more than 50 µm. Within these structures, phosphorous seems to be depleted (Figure 4). Micro-CT image analysis of the set cements indicate a gradual increase in measured average greyscale values with increased concentration of baghdadite in the cement. Increased substitution of beta-TCP with baghdadite in the brushite cement formulation resulted in increased radiopacity of the set cements, with the radiopacity HU values increasing linearly with increased baghdadite substitution (R^2^ > 0.99) as shown in Figure 5a. BC, BCB5, BCB10, and BCB20 showed 2610 ± 130 HU, 2930 ± 50 HU, 3160 ± 80 HU, and 3910 ± 130 HU, respectively, when scanned at 70 keV x-ray energy. All cement groups showed statistically significant differences from one another. X-ray images comparing the brushite and baghdadite-substituted brushite cements with various thicknesses of aluminum (Figure 5b) also demonstrated increased radiopacity with increased baghdadite concentration. BC, BCB10, BCB20, and BCB50 showed equivalent aluminum thicknesses of 1.24 ± 0.2 mm, 1.53 ± 0.2 mm, 1.56 ± 0.1 mm, and 2.19 ± 0.1 mm, respectively. This higher radiopacity also leads to an improved visibility of cement implants within a bony structure as demonstrated by filling bore-holes in a porcine mandible with cement (Figure 6).

### 3.4. In Vitro Cytocompatibility

**Figure 7:**
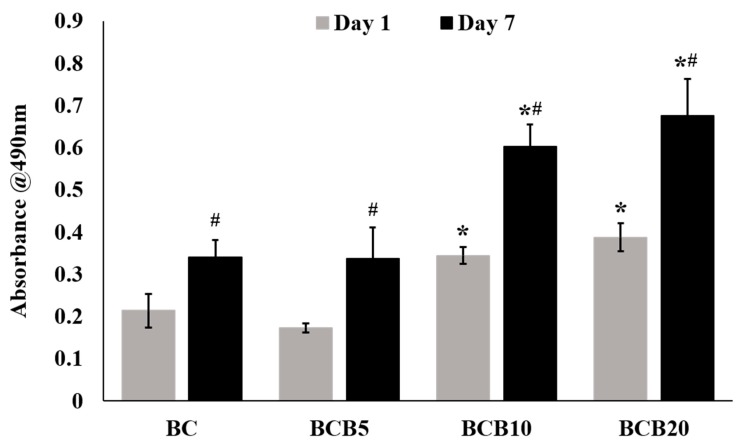
MTS assay absorbance values recorded for primary human osteoblasts cultured in media conditioned with 200 mg/mL of BC, BCB5, BCB10, and BCB20 after 1 day and 7 days of cell culture. #: *p* < 0.05 vs. day 1 absorbance values of the corresponding material; *: *p* < 0.05 vs. BC and BCB5 at the corresponding cell culture time point.

The pH and ion content of each of the culture media conditioned with BC, BCB5, BCB10, and BCB20 are listed in Table 3. The pH of the culture media conditioned with BC, BCB5, BCB10, and BCB20 were measured to be 6.47, 6.57, 6.73, and 7.02, respectively. The media conditioned with BCB5, BCB10, and BCB20 indicate the presence of silicon ions ranging 64.7 to 69.9 ppm, whereas no silicon ions were detected in BC-conditioned media. Culture media conditioned BCB10 and BCB20 showed almost half the phosphorus ion levels (362.9 ppm and 349.1 ppm, respectively) compared to those conditioned with BC (709.5 ppm). No zirconium ions were detected in any of the culture medium solutions. Culture medium conditioned with BCB10 showed the highest calcium concentration out of the groups tested and showed calcium levels of 34.4 ppm. Figure 7 shows all cement-conditioned media samples showed statistically significant increases in absorbance values between day one and day seven within the respective cement groups. The absorbance at 490 nm for BCB10 samples were significantly higher than BC and BCB5 samples for both the day one and day seven time points of primary HOB culture. Similarly, the absorbance values recorded for BCB20 samples were significantly higher than BC and BCB5 for days one and seven. No statistically significant differences were observed for all time points between BC and BCB5, and no statistically significant differences were observed between BCB10 and BCB20 after one and seven days of HOB culture. 

## 4. Discussion

Conventional brushite cements are formed via a dissolution-precipitation mechanism of the slightly basic β-tricalcium phosphate particles and acidic monocalcium phosphate monobasic in equimolar amounts. Previous studies have developed and characterized modified brushite cements, by using Mg-, Zn-, and Sr- substituted β-TCP as the primary reactant, resulting in improved biological activity of the materials [38,39,40]. Other studies have reported the effect on the brushite cements upon additional incorporation of calcium silicate reactants [41,42]. This is the first study to modify the brushite reaction by way of substituting the β-TCP phase with baghdadite, a calcium silicate ceramic with zirconium incorporated into its stoichiometry, recently shown to possess excellent bone regeneration properties in vitro and in vivo, in comparison to calcium phosphate ceramics. Similar to β-TCP, baghdadite powder is also slightly basic, and is shown in this study to dissolve in the cement reaction and become actively incorporated into the brushite structure, rather than acting as a passive non-reacting additive. The stoichiometry of the setting reactions for cements with pure β-TCP and pure baghdadite are shown in Equations (1)–(3):

Ca_3_(PO_4_)_2_ + Ca(H_2_PO_4_)_2_ + 8H_2_O → 4CaHPO_4_·2H_2_O (1)

Ca_3_ZrSi_2_O_9_ + Ca(H_2_PO_4_)_2_ + 3H_2_O → 2CaHPO_4_·2H_2_O + Ca_2_ZrSi_2_O_8_ (2)

Ca_3_ZrSi_2_O_9_ + 3Ca(H_2_PO_4_)_2_ + 9H_2_O → 6CaHPO_4_·2H_2_O + ZrSi_2_O_6_ (3)

While the reaction of cement BC with pure β-TCP results in the quantitative formation of brushite due to an equimolar ratio of both reactants, the reaction of baghdadite with MCPA forms both brushite and a calcium depleted ceramic composed of calcium, zirconium, and silicate (2) or a zirconium silicate (3), depending on the baghdadite: MCPA ratio. Since XRD analysis indicated even for the highest baghdadite content in BCP100 only small diffraction peaks for the baghdadite raw powder, it can be assumed that baghdadite reacts practically in a quantitative manner according to Equations (2) and (3). Since also no further diffraction peaks could be found, it is also likely that the byproducts formed must have an amorphous nature. As the elemental mapping in EDX analysis (Figure 4) showed the presence of zirconium, silicon, and calcium within the same local area, it is also likely that only a partial depletion of baghdadite has occurred according to Equation (2) rather than the formation of a pure zirconium silicate compound. The simultaneously formed brushite phase showed no crystallographic changes due to the addition of baghdadite as cement reactant as demonstrated by analyzing the crystal lattice parameters by Rietveld refinement analysis. The microstructure of the set cements with increased baghdadite substitution also still shows the characteristic plate-like structures of brushite crystals, however, with a reduced crystal size and a second phase of closely packed particles sub-micron particles. 

The reduction in β-TCP + MCPM reaction due to increased baghdadite substitution of β-TCP did not negatively affect the mechanical integrity of the final set brushite cements, but rather enhanced its mechanical properties for a baghdadite substitution of up to 10–20wt.%, further suggesting the active role of baghdadite in the cement reaction. The final cement compressive strength attained in our brushite and baghdadite-modified brushite is lower than those reported elsewhere [15], primarily due to the use of a lower powder-to-liquid ratio of 3.0 g/mL. In this study, we intentionally used this lower PLR to sufficiently investigate the effect of baghdadite substitution in the final brushite cement production, as opposed to other studies that typically use ratios higher than 3.3 g/mL. For example, mixing the BCB20 formulation at 3.3g/mL did not allow enough time for sufficient mixing of the components and subsequent transfer into molds, and that a PLR of 3.0 g/mL was optimal for the preparation of all the cements investigated in this study, and was maintained for all groups for scientific validity for observing the effect of baghdadite in the brushite cement reaction. Although lower PLR typically results in lower mechanical strength of set cements [18,20,43], lower PLR also allows for good homogenous mixing of the powder components and reduces the potential effect of manual mixing on the final obtained results. The baghdadite-substituted brushite cements still achieve compressive strengths of up to 21 MPa, which is within range of the compressive strengths reported for cancellous bone. In addition, baghdadite substitution can readily be incorporated into previously published strategies that have successfully enhanced the strength of brushite cement through other means such as controlling particle size and use of alternate additives into the cement reaction [15,44].

The central hypothesis of our study was an enhancement of radiopacity of the cements due to baghdadite substitution. Radiopacity of bone cements is an important property to consider when monitoring the cement and the surrounding bone during and after surgery. This was clearly verified by measuring X-ray absorption of samples and determining the aluminum equivalent thickness according to ISO4049 [36], which nearly doubles for 50wt.% substitution. In addition, micro-CT images also indicate approximately 50% increase in the measured radiopacity in Hounsfield units when exposed to X-ray at 70 keV energy. Though the Hounsfield units obtained depends on the x-ray energy, the values obtained from CT scans is a useful quantitative tool in comparing the radiopacity of materials under clinically relevant conditions [45]. An advantage of our approach compared to current strategies to enhance radiopacity of bone cements by using hard bioinert particles such as barium sulfate and zirconia is that these inert particles are simply embedded into the brittle cement structure and subsequently can weaken its mechanical properties. In this study, we were able to achieve simultaneous increases in radiopacity and mechanical strength through baghdadite-substitution into brushite cement due to the cementitious reactivity of the baghdadite component under acidic conditions.

Regarding cytocompatibility issues, baghdadite substitution positively influenced the proliferation activity of primary human osteoblast. Such increased proliferation effect is most likely a function of both the increased bioactivity of baghdadite when compared to calcium phosphate-based materials as reported previously [31], as well as the increased starting pH of the culture media conditioned with baghdadite-substituted brushite compared to that conditioned with unmodified brushite cement. The pH of the culture media used was affected by the amount of baghdadite substituted into the brushite, with culture media conditioned with BCB20 showing pH of ~7 compared to ~6.5 for pure brushite. It is reported in the literature that brushite cement is acidic in aqueous environments [5,22], and though brushite cements have been shown to demonstrate good in vivo bone regeneration [5,6], there is very little in vitro analysis done on brushite cements due to the inherent inability to continuously ‘flush out’ the acidity in a typical in vitro experiment. Low pH has been shown to detrimentally affect osteoblasts [24], and so by incorporating baghdadite into brushite cements we would be able to reduce the effect of acidity in the implant region for bone cement applications, especially during the early setting phase when the bodily fluids are closely interacting with the applied cement. A second effect is related to the reduced phosphate content of the culture medium. Here, previous studies have revealed that brushite cements can increase the phosphate concentration of cell culture medium by the factor of 20–30 during cell culture [46], whereas phosphate concentrations exceeding a certain level have a detrimental effect on cell viability in vitro. Baghdadite substitution in brushite cements showed a strong reduction in phosphate release and may hence also be responsible for the observed better cytocompatibility of the materials. 

## 5. Conclusions

This study has demonstrated that baghdadite substitution of β-TCP is a useful strategy to improve the properties of brushite cements for bone defect filling applications by its radiopacity and in vitro HOB proliferation, without significantly compromising its mechanical properties for up to 50 wt% substitution. This strategy may be used concurrently with other reported protocols to further optimize the formulation of brushite-based cements, as well as potentially other mineral based cements such as apatite- and calcium sulfate-based cements.

## Figures and Tables

**Table 1:** Composition of the powder reactants per gram for each cement formulation investigated in this study.

Baghdadite/[Baghdadite + β-TCP] (wt%)	β-TCP (g)	MCPA (g)	Baghdadite (g)	Code
0	11.04	8.31	0.000	BC
5	10.48	8.31	0.56	BCB5
10	9.92	8.31	1.12	BCB10
20	8.82	8.31	2.24	BCB20
30	7.70	8.31	3.36	BCB30
50	5.52	8.31	5.52	BCB50
100	0	8.31	11.04	BCB100

**Table 2:** Brushite lattice plane reflex positions adjusted to the corundum reference for sample BCB50%.

Lattice Plane	Lattice Parameter: Diffraction Angle in 2θ, (Distance d in nm)	
	Theoretical	Measured
0 2 0	11.65 (7.59)	11.62 (7.61)
1 2 −1	20.95 (4.24)	20.95 (4.24)
1 1 −2	29.28 (3.05)	29.28 (3.05)
1 2 1	30.53 (2.93)	30.52 (2.93)
2 0 0	34.15 (2.62)	34.13 (2.62)

**Table 3:** pH and ion content of culture media conditioned with BC, BCB5, BCB10, and BCB20. All ion concentration measurements in parts per million (ppm). n.d.: not detectable.

pH/Ion Element	BC	BCB5	BCB10	BCB20
pH	6.47	6.57	6.73	7.02
Calcium	27.9	29.7	34.4	27.2
Sodium	3429.9	3325.8	3207.5	3220.6
Phosphorus	709.5	560.9	362.9	349.1
Silicon	n.d.	64.7	69.9	65.9
Zirconium	n.d.	n.d.	n.d.	n.d.
